# Low knowledge of newborn danger signs among pregnant women in Papua New Guinea and implications for health seeking behaviour in early infancy – findings from a longitudinal study

**DOI:** 10.1186/s12884-022-05322-6

**Published:** 2023-01-26

**Authors:** Donya Eghrari, Michelle J. L. Scoullar, Alyce N. Wilson, Elizabeth Peach, Arthur Elijah, Pele Melepia, Hadlee SupSup, Lisa M. Vallely, Peter M. Siba, Elissa C. Kennedy, Joshua P. Vogel, Caroline S. E. Homer, Leanne J. Robinson, Freya J. I. Fowkes, William Pomat, Brendan S. Crabb, James G. Beeson, Christopher J. Morgan

**Affiliations:** 1 Burnet Institute, Melbourne, Australia; 2 University of Melbourne, Melbourne, Australia; 3 Burnet Institute, Kokopo, Papua New Guinea; 4 University of Papua New Guinea, Port Moresby, Papua New Guinea; 5 Port Moresby General Hospital, Port Moresby, Papua New Guinea; 6 East New Britain Provincial Health Authority, Kokopo, Papua New Guinea; 7 Papua New Guinea Institute of Medical Research, Goroka, Papua New Guinea; 8 The Kirby Institute, University of New South Wales, Sydney, Australia; 9 Australian Institute of Tropical Health and Medicine, James Cook University, Townsville, Australia; 10 Center for Health Research and Diagnostics, Divine Word University, Madang, Papua New Guinea; 11 Monash University, Melbourne, Australia; 12 Murdoch Children’s Research Institute, Melbourne, Australia; 13 Jhpiego, a Johns Hopkins University Affiliate, Baltimore, USA

**Keywords:** Knowledge, Newborn, Danger signs, Pregnant women, Care seeking, Antenatal and postpartum education

## Abstract

**Background:**

Globally, 2.5 million babies die in the first 28 days of life each year with most of these deaths occurring in low- and middle-income countries. Early recognition of newborn danger signs is important in prompting timely care seeking behaviour. Little is known about women’s knowledge of newborn danger signs in Papua New Guinea. This study aims to assess this knowledge gap among a cohort of women in East New Britain Province.

**Methods:**

This study assessed knowledge of newborn danger signs (as defined by the World Health Organization) at three time points from a prospective cohort study of women in East New Britain Province, factors associated with knowledge of danger signs after childbirth were assessed using logistic regression. This study includes quantitative and qualitative interview data from 699 pregnant women enrolled at their first antenatal clinic visit, followed up after childbirth (*n* = 638) and again at one-month post-partum (*n* = 599).

**Results:**

Knowledge of newborn danger signs was very low. Among the 638 women, only 9.4% knew three newborn danger signs after childbirth and only one knew all four essential danger signs defined by Johns Hopkins University ‘Birth Preparedness and Complication Readiness’ Index. Higher knowledge scores were associated with higher gravidity, income level, partner involvement in antenatal care, and education.

**Conclusion:**

Low levels of knowledge of newborn danger signs among pregnant women are a potential obstacle to timely care-seeking in rural Papua New Guinea. Antenatal and postnatal education, and policies that support enhanced education and decision-making powers for women and their families, are urgently needed.

**Supplementary Information:**

The online version contains supplementary material available at 10.1186/s12884-022-05322-6.

## Key points

Patient and public involvement

- Patients and members of the public were not involved in the design, or conduct, or reporting, or dissemination plans of the research. The study, however, was designed and conducted in close consultation with East New Britain Provincial Health authorities as part of an ongoing research collaboration, called the *Healthy Mothers Healthy Babies* program, in which we are working together to improve maternal and child health outcomes.

What is already known on this topic

- Early recognition of newborn danger signs plays an important role in initiating timely care seeking. There is minimal evidence about women’s knowledge of newborn danger signs in Papua New Guinea.

What this study adds

- Knowledge of newborn danger signs among women was low in pregnancy and after childbirth, and likely insufficient for timely newborn care-seeking. Women with higher knowledge scores were those with a previous pregnancy, higher income and a male partner who attended antenatal care with them.

How this study might affect research, practice or policy

- This study highlighted the need for greater efforts in targeted perinatal education, particularly for first time parents, and ensuring education is effectively received and understood by pregnant women and their families*.* There is an urgent need to review policies that support decision-making powers for women and their families, as well as enhance antenatal and postnatal education.

## Introduction

Major gains have been made globally in reducing child mortality over the past three decades, with a halving of the mortality rate for children under five [1]. However, the rate of decline for neonatal mortality has been slower and neonatal deaths now make up almost half (47%) of all deaths under five, with the vast majority of these (99%) occurring in low– and middle–income countries (LMIC) [1]. In order to reach target 3**∙**2 of the Sustainable Development Goals (SDG) — reduction of neonatal mortality to 12 deaths or less per 1,000 live births by 2030 [2] — a major focus is needed on reducing risk factors associated with newborn mortality and improving access to quality family–centred care.

Newborn danger signs are symptoms and clinical signs that commonly indicate severe sickness in a baby at birth or in the immediate postnatal period [3, 4]. Compared to later in infancy, recognition of newborn danger signs is often more difficult as newborn illness can present with non–specific signs and symptoms [5], and timely recognition is critical as newborn illnesses can progress very rapidly [6]. Initiation of the healthcare seeking cascade is highly dependent on the primary caregiver’s (often the mother) knowledge and recognition of neonatal danger signs [7], and a delay at this stage can increase the risk of a poor newborn outcome [8, 9]. Timely recognition of newborn danger signs can be more critical in remote settings where there may be delay in reaching healthcare facilities due to distance and limited travel means [10].

Papua New Guinea (PNG), a LMIC in the Asia–Pacific region, has a population of over 8.5 million [11] and has one of the highest neonatal mortality rates in the region at 20 deaths per 1,000 live births [12, 13]. The PNG National Health Plan (2012–2020) considers neonatal health and survival as a priority area [14] and aims to improve health outcomes for newborns through improved maternal health services, facility-based births, and enhanced capacity to provide life–saving support to the newborns. However, despite these efforts, the SDG target for NMR is unlikely to be achieved sooner than 2050 in PNG [15].

Vital to this work is evidence on women’s knowledge of newborn danger signs, yet very limited evidence is available in PNG, and the broader Pacific region [16]. Two studies from PNG have previously investigated knowledge of danger signs, one reported women’s knowledge of maternal, but not newborn, danger signs in pregnancy and childbirth [17] (among 482 women), and the other reported on a cross–sectional survey that included mother’s knowledge of newborn danger signs evaluated within two years of childbirth [18] (among 482 women). Here we provide additional insights, reporting knowledge of newborn danger signs among women at three important timepoints: early pregnancy, after childbirth, and one month postpartum. The study included a longitudinal cohort of pregnant women in East New Britain Province (ENBP) who were enrolled and followed from first antenatal clinic visit through to childbirth and the post-partum period.

## Methodology

### Setting, population and sampling

The *Healthy Mothers, Healthy Babies (HMHB)* [19] research program was established in East New Britain Province (ENBP) in 2015 and consists of several primary studies and implementation projects all working towards identifying determinants of poor maternal and newborn health [20–22]. ENB is part of a large island in PNG’s north–east, whose population of approximately 400,000 live in a mix of highland and coastal villages, with some small towns [23]. The HMHB Prospective Observational Longitudinal Cohort Study, conducted between March 2015 and December 2018, recruited 699 pregnant women at their first antenatal clinic (ANC) with follow up after childbirth, and again at 1, 6, and 12 months postpartum; data from the first three time points (ANC1, childbirth and 1 month postpartum) were used for this study. Eligible women were those aged 16 years or more, living in the health facility catchment, and who agreed to participate and provided written informed consent. Women were enrolled by using a dice to select randomly from clinic attendees. Of the 699 enrolled, 638 (91%) were followed to childbirth, and 599 (86%) to one–month post–partum.

### Study procedures

Interviews using a structured questionnaire, including both quantitative and qualitative components, were conducted in private settings at the ANC clinic, health facility after childbirth and the woman’s home (after childbirth and at one month postpartum). Questions covered demographics, obstetric history, experience of health care, health knowledge, and preferences for care, in addition to physical examinations and collection of biological samples. Interviews were administered by trained local research staff in local languages, guided by a questionnaire on an electronic tablet. Participants received routine care in accordance with PNG national guidelines, including facilitation of follow–up of any health issues disclosed in the interview. Knowledge of newborn danger signs was assessed by asking women to name (in their local language) any signs or symptoms in their newborn that would prompt them to seek urgent assessment for their baby (Supplementary Table 1). Interviewers coded these against a list based on World Health Organization (WHO) guidance on newborn danger signs [5]. Follow–up questions allowed for clarification or additional detail.

### Data analysis

Data were de–identified and analysed with Stata 15**∙**0 for each time point – antenatal care, after childbirth and one month postpartum. Responses to knowledge questions were calculated as simple proportions. Secondary analysis drew on international literature to describe a minimum effective knowledge level, based on the type and the number of danger signs reported. Data were analysed by the research team, referencing the eight newborn danger signs described by the WHO [5] and four signs identified by Johns Hopkins University’s Birth Preparedness and Complication Readiness (JH BPCR) Index [3]. A composite outcome measure was created and defined as: knowledge of three or more danger signs. Bi–variate and multivariable logistic regression models assessed the association between this composite outcome measure and possible determinants including: age, marital status, maternal birth province, travel time to clinic, monthly household expenditure, gravidity, education and employment status of the woman and her partner, and partner involvement at ANC (defined as partner being present in the clinic room while health worker performed assessment). Variables used in the multivariable model, to adjust for possible confounders, were decided through discussion within the research team, based on known associations in published literature. Variables with clear co–linearity were excluded. Crude and adjusted odds ratios with 95% Confidence Interval (CIs) and *p*–values assessed the direction and strength of the statistical association.

## Results

The sociodemographic and obstetric profile of mothers and their partners is shown in Table 1. The study assessed interview data from 699 pregnant women enrolled at their first ANC visit, followed up in the early postpartum period after childbirth (*n* = 638) and again at one-month post-partum (*n* = 599). More than half of women were aged over 25 (417/692, 60**∙**2%). The median monthly household expenditure was approximately 150 Kina (~ $42USD in October 2021). Approximately 17**∙**7% (123/694) of women’s male partners were present at first ANC visit. The majority of women were multigravida (522/697, 74**∙**9%), married or cohabiting (663/697, 95**∙**1%), and unemployed (531/699, 76**∙**0%).

**Table 1:** Socio–demographic and obstetric variables of women at baseline (first antenatal care visit)

VARIABLE		n (%) *N* = 699
Sociodemographic details for enrolled women		
Enrolment Clinic	St Mary’s Hospital Vunapope	184 (26∙3)
	Nonga General Hospital	83 (11∙9)
	Kerevat Rural Hospital	125 (17∙9)
	Napapar Health Centre	158 (22∙6)
	Paparatava Health Centre	149 (21∙3)
Clinic admin	Government	208 (29∙8)
	Non–government (Catholic Health)	491 (70∙2)
Age, in years ^m1^	Median {IQR}, range	26 {22–30}, 16–49
	16–24	275 (39∙7)
	25–34	334 (48.3)
	35 +	83 (11∙9)
Highest level of education completed ^m2^	Primary (Grade 8 or less)	325 (46∙6)
	High school (grade 9,10)	177 (25∙4)
	Secondary (grade 11,12)	50 (7∙2)
	Vocational or Tertiary	146 (20∙9)
Employment status	Not employed	559 (80.0)
	Employed	140 (20.0)
Province of birth	East New Britain	578 (82∙7)
	Other Province	121 (17∙3)
Religion ^m3^	Catholic	345 (53.4)
	United	225 (34.8)
	Other	76 (11.7)
Marital status ^m4^	Married or cohabiting	663 (95.1)
	Single, separated or widowed	34 (4∙9)
Household monthly expenditure (in Kina) ^m5^	Median {IQR}	150 {50–300}
	150 Kina or less	354 (50∙6)
Time to clinic in minutes (as reported by woman – walk or car) ^m6^	Median {IQR}	30 {10–45}
	25 min or less	253 (49.0)
	Over 25 min	263 (51.0)
Partner details		
Partner's highest level of education ^m7^	Primary (Grade 8 or less)	193 (30.5)
	High school (grade 9,10)	147 (23.2)
	Secondary (grade 11,12)	81 (12.8)
	Vocational or Tertiary	212 (33.5)
Partner's employment status ^m8^	Not formally employed	269 (39.6)
	Employed in paid work	411 (60.4)
Partner attending ANC1 ^m9^	No (incl. No but would like to be)	571 (82.3)
	Yes	123 (17∙7)
Maternal Health Parameters at 1st Antenatal Clinic		
Gravidity ^m10^	Primigravidae	175 (25∙1)
	Multigravidae	522 (74∙9)

m#: missing data (%)

[m1: 7(1∙0); m2: 1(0∙1); m3: 53(7∙6); m4: 2(0∙3); m5: 36(5∙2); m6: 183(26∙2)

m7: 66(9∙4); m8: 19(2∙7); m9: 5(0∙7); m10: 2(0∙3)]

### Frequency of clinically–significant newborn danger signs reported

Proportions of women reporting important danger signs were quantified at three time points (Table 2). At the antenatal visit, 31∙1% (215/692) of women could not name any danger signs, decreasing to 15% (87/581) at one–month post–partum. At the visit after childbirth, only one woman named all four key signs in the JHBPCR; 9**∙**8% (61/625) named three or more danger signs, and 72**∙**3% (452/625) named one or two danger signs (Table 3). Fever and ‘not feeding’ were the most commonly reported danger signs across all time points. Very few women named any of the four key signs identified in the JHBPCR as those essential to life–saving care–seeking, with breathing difficulties being the most common danger sign women reported (reported by 14**∙**4% [90/625] at the first postpartum visit). Some trends over the three time points were apparent; more women could name one or more danger signs at later time points. A higher proportion of women named fever after childbirth and one–month post–partum than at first ANC.

**Table 2:** Newborn danger signs identified by women at the three different time–points

	**At First ANC Visit** ***n* = 692**^**m1**^	**After Birth** ***n* = 625**^**m2**^	**One month postpartum** ***n* = 581**^**m3**^
**WHO Newborn Danger Signs**	Frequency (%, 95% CI)	Frequency (%, 95% CI)	Frequency (%, 95% CI)
No danger signs named	215 (31.1, 27.6 -34.7)	112 (17.9, 15 -21.2)	87 (15.0, 12.2 -18.1)
Difficulty/fast breathing^a^	81 (11.7, 9.4 -14.3)	90 (14.4, 11.7 -17.4)	73 (12.6, 10 -15.5)
Fits / Convulsions^a^	31 (4.5, 3.1 -6.3)	24 (3.8, 2.5 -5.7)	35 (6.0, 4.2 -8.3)
Lethargy (not moving)^a^	20 (2.9, 1.8 -4.4)	14 (2.2, 1.2 -3.7)	12 (2.1, 1.1 -3.6)
Baby too small/not growing^a^	9 (1.3, 0.6 -2.5)	10 (1.6, 0.8 -2.9)	7 (1.2, 0.5 -2.5)
Baby too hot/fever	361 (52.2, 48.4 -55.9)	421 (67.4, 63.5 -71)	430 (74.0, 70.2 -77.5)
Baby not feeding	129 (18.6, 15.8 -21.7)	138 (22.1, 18.9 -25.5)	93 (16.0, 13.1 -19.2)
Body unusually cold	69 (10.0, 7.8 -12.4)	71 (11.4, 9 -14.1)	42 (7.2, 5.3 -9.6)
Yellow skin or eyes (Jaundice)	40 (5.8, 4.2 -7.8)	37 (5.9, 4.2 -8.1)	22 (3.8, 2.4 -5.7)
Severe chest in–drawing	0	0	0

m1, 7 missing response from 699 women; m2, 13 missing resonse from 638 women; m3, 18 missing response from 599 women

^a^Key danger signs in the newborn as defined by JH BPCR(11)

Full list derived from World Health Organisation 2017(6)

**Table 3:** Adequacy of pregnant and postpartum women’s knowledge of newborn danger sings

Number of WHO Newborn Danger Signs reported	Number of women with various levels of knowledge n(%)		
	Antenatal Care *n* = 692	Childbirth *n* = 625	One month postpartum *n* = 581
No danger signs reported	215 (31.1)	112 (17.9)	87 (15.0)
1 or 2 danger signs reported	419 (60.5)	452 (72.3)	455 (78.3)
≥ 3 danger signs reported	58 (8∙4)	61 (9.8)	39 (6∙7)
All 4 JH BPCR key signs^a^	0	1 (0∙2)	0

^a^JH BPCR 4 key danger signs: difficulty/fast breathing, fits/convulsions, lethargy (not moving), baby too small/not growing

### Characteristics associated with women’s knowledge of newborn danger signs after childbirth

Associations with knowledge of three clinically important danger signs shortly after childbirth were analysed using bivariate and adjusted multivariable logistic regression models (Table 4). Age, in the bivariate model only, and gravidity, in bivariate and adjusted model, both showed associations with knowledge at childbirth; where women with a history of previous pregnancies had 3**∙**86–fold greater odds of reporting three or more danger signs compared to first time pregnant mothers (95% CI 1**∙**25 – 11**∙**89, *p* = 0**∙**019). Monthly expenditure (as a proxy for wealth) had a suggestive association with knowledge across all time points. At childbirth women with monthly expenditures above 300 Kina had a 3∙08–fold (95% CI 1∙36 – 7∙00, *p* = 0∙007) increased odds of naming three or more danger signs, however this relationship weakened once confounders (such as women’s employment and partners’ education) were included in the multivariable model (adjusted OR 2∙26, 95% CI 0∙9 – 5∙64, *p* = 0∙081). It was only at one-month post-partum that associations were found with other factors such as education, greater age, or accompaniment by a partner at ANC visit (Supplementary Table 6).

**Table 4:** Association between women naming ≥ 3 newborn danger signs and potential determinants of knowledge, at interviews shortly after childbirth

Sociodemographic and Obstetric characteristics	Crude analysis	Adjusted analysis
	**OR (95% CI); *p*-value**	**OR (95% CI); *p*-value**
**Enrolment Clinic**		
Vunapope (REF)	REF	REF
Nonga	0.44 (0.16–1.19); 0.106	0.58 (0.2–1.64); 0.301
Keravat	0.53 (0.24–1.19); 0.125	0.47 (0.2–1.12); 0.09
Napapar	0.46 (0.22–0.98); 0.043	0.52 (0.22–1.2); 0.126
Paparatava	0.5 (0.23–1.05); 0.066	0.63 (0.28–1.4); 0.257
**Clinic administration**		
Government (REF)	REF	
Church Health Facility	1.34 (0.72–2.5); 0.357	
**Age, years**		
16–24 (REF)	REF	REF
25–34	**2.09 (1.1–3.97); 0.025**	1.28 (0.63–2.6); 0.503
35 years or older	**3.05 (1.31–7.07); 0.009**	1.58 (0.62–4.01); 0.334
**Highest level of education completed**		
Primary school (Grade 8 or less)	REF	REF
High school (Grade 9, 10)	1.2 (0.62–2.34); 0.583	0.98 (0.47–2.05); 0.957
Secondary (Grade 11,12)	1.33 (0.48–3.69); 0.582	1.55 (0.52–4.61); 0.43
Vocational or Tertiary	1.52 (0.78–2.97); 0.221	1.19 (0.58–2.47); 0.63
**Maternal employment status**		
Not employed	REF	
Employed	1.49 (0.81–2.75); 0.196	
**Province of Birth**		
East New Britain	REF	REF
Other Province	1.67 (0.9–3.12); 0.106	1.31 (0.66–2.61); 0.443
**Religion**		
Catholic	REF	
United	0.81 (0.43–1.5); 0.5	
Other	1.8 (0.87–3.71); 0.112	
**Household monthly expenditure in Kina**		
Poorest quintile (REF)	REF	REF
50–150	1.56 (0.66–3.67); 0.313	1.28 (0.51–3.16); 0.6
150–300	1.56 (0.65–3.71); 0.319	1.44 (0.58–3.6); 0.436
> 300	**3.08 (1.36–7); 0.007**	2.26 (0.9–5.64); 0.081
**Time to clinic in minutes**		
25 min or less(REF)	REF	
More than 25 min	0.83 (0.45–1.54); 0.564	
**Partner's highest level of education**		
Primary school (Grade 8 or less)	REF	
High school (Grade 9, 10)	0.49 (0.21–1.17); 0.108	
Secondary (Grade 11,12)	0.48 (0.16–1.45); 0.192	
Vocational or Tertiary	1.17 (0.62–2.2); 0.634	
**Partner's employment status**		
Not employed/house duties	REF	REF
Employed	1.38 (0.78–2.42); 0.264	1.07 (0.57–2.01); 0.833
**Partner present at ANC**		
No not present	REF	REF
Yes at ANC	1.37 (0.73–2.59); 0.327	1.37 (0.7–2.68); 0.36
**Gravidity**		
Primigravida	REF	REF
Multigravida	**3.86 (1.52–9.82); 0.005**	**3.86 (1.25–11.89); 0.019**

Marital status (Married or cohabiting/ Single/separated/widowed) was omitted because of collinearity

## Discussion

These findings show that knowledge of clinically significant danger signs among mothers in East New Britain is inadequate for informed decision–making on care seeking in the case of serious neonatal illness. At childbirth, only one woman knew all four key danger signs that help identify life–threatening illness [3] and 17% could not report any danger signs. This is consistent with findings of studies in other resource–constrained settings such as India, Nigeria, Ethiopia, and Uganda [24–29] which identified inadequate maternal knowledge regarding newborn danger signs.

This study did not assess actual care–seeking in response to danger signs, however other studies have found that knowledge of one WHO–recognised danger sign can prompt a caregiver to initiate care–seeking [26]. Among WHO–defined newborn danger signs, fever was most frequently reported as known by women in ENBP — mirroring prior findings from PNG [18] and other settings [26, 30, 31] — followed by difficulty with feeding. Both are important danger signs, although many life–threatening newborn conditions do not present with fever [5], and this sign is not included in the JHBPCR core set. It is perhaps unsurprising that fever was mentioned by many mothers, given that febrile illness – especially related to malaria – has a prevalence in PNG 3∙5 times higher than the global average [32, 33]. However, it is concerning that other important signs in the newborn period, such as convulsions, breathing difficulties and low body temperature, were named infrequently by study participants. These are signs that are possibly not learned through general experience, as they occur less commonly, but rather need active education to raise awareness of them.

Women’s awareness of danger signs may be derived more from lived experience than from health education; noting that the number of women naming common symptoms, such as fever and cough, did increase from childbirth to one–month post–partum. This is also supported by our regression analysis, which found that at the childbirth time point, greater knowledge had a stronger association with previous experience of pregnancy, than with other factors such as education. It was only in interviews at one–month post–partum, the conclusion of the neonatal period, that we found associations with other factors such as education, greater age, or accompaniment by a partner at ANC visit (as also reported by Zaman et al. (2018) [34]).

Previous studies in PNG have suggested the need for health education to incorporate recognition of maternal and newborn danger signs [35]. These studies, in addition to our findings, highlight the need for greater efforts in targeted perinatal education, especially for first time parents and during routine postnatal care. Others have highlighted the potential of high–quality ANC education [36] and postnatal care [37] to empower women to take a more proactive role in seeking healthcare for their newborn. There is also a high rate of unplanned pregnancy and low use of family planning methods in this population [21]. Given previous pregnancy experience was associated with greater knowledge in our data, there may be opportunities for experienced mothers to share and teach first–time mothers in peer–to–peer or group education models [38]. Education at a postnatal pre–discharge discussion (for babies born in health facilities) has improved women’s knowledge of newborn danger signs in other settings [31, 39]. In responding to our findings, the ENB Provincial Health Authority supported renewed efforts to improve knowledge of maternal and newborn danger signs through a postnatal care implementation research project [40], capitalising on the postnatal period as a feasible time point for the delivery of tailored postnatal education.

Adequate knowledge and the ability to recognise danger signs may not be enough to always initiate care seeking behaviour. Family income and financial resources can play a role in maternal and child health service utilisation in PNG [41]. Our findings showed that wealthier women (using our proxy expenditure measure) had greater odds of knowing three or more danger signs, which may be suggestive of greater autonomy and household decision making powers for the mother [42–44]. While PNG has a free primary healthcare policy, it cannot always be applied due to facility financial constraints. Decision making powers do not always rest with the mother but can be influenced by others (such as the male partner or older women), it may be that wealthier households allow greater prioritisation and allocation of funds to the mother for out–of–pocket expenses, increasing her likelihood of service utilisation, especially in the case of illness [45].

A complete response to our findings must necessarily entail broader strengthening of access to, and receipt of quality newborn care, alongside efforts to improve antenatal and postnatal education and service delivery, reduced costs of access and a continued emphasis on the role of the partner [46–50], family and the wider community [51, 52]. In addition, there are currently no standardised indicator sets used in the assessment of newborn danger sign knowledge and definitions of adequate knowledge in studies and reference documents vary widely. Whilst knowledge of three or more danger signs is the most frequently reported criteria in the literature [31, 53, 54] a standardised indicator set could be a useful tool for determining adequate knowledge required to optimise newborn health outcomes.

### Strengths and limitations

Strengths of this study include the use of a longitudinal cohort design allowing analysis of knowledge changes over the critical period of pregnancy, childbirth, and early postpartum periods. Limitations include that recruitment was limited to women who were already attending a healthcare facility for ANC, which covered an average of 78% of all pregnant women presenting to a health facility in ENBP during recruitment [32]. The knowledge of newborn danger signs among women who did not attend ANC is unknown. We used the reporting of newborn danger signs, without use of prompts, as an indicator of knowledge, recognising that this is not an absolute measure of knowledge or action that may be taken, and we did not assess healthcare seeking behaviour in response to knowledge.

## Conclusion

Caregivers’ knowledge and recognitions of danger signs in the newborn period plays a vital early step towards appropriately seeking care for severe illness. Our findings demonstrate that knowledge of key newborn danger signs in East New Britain is critically low, but that knowledge does increase from early pregnancy to childbirth and that women with previous pregnancies have higher levels of knowledge. This likely reflects a learning that occurs through antenatal care and that of lived experience. These findings indicate a need to strengthen ANC and postnatal education for parents, particularly first-time parents, alongside other efforts to improve quality and access to care, and empowering partners and communities in efforts to promote newborn survival in PNG.

## Supplementary Information

**Additional file 1.**

## Data Availability

The datasets generated and/or analysed during the current study are not publicly available due to potential confidentiality concerns. Additional information can be made available from the Scientific Integrity Officer at Burnet Institute, (admin@ burnet.edu.au), on reasonable request.

## Abbreviations

PNG: Papua New Guinea
ENB: East New Britain
ENBP: East New Britain Province
LMIC: Low– and middle–income countries
SDG: Sustainable Development Goals
ANC: Antenatal Care
HMHB: *Healthy Mothers, Healthy Babies*
MNH: Maternal Newborn Health
WHO: World Health Organisation
JH BPCR: Johns Hopkins University’s Birth Preparedness and Complication Readiness
CI: Confidence Interval
