# Supplementary material for: Low knowledge of newborn danger signs among pregnant women in Papua New Guinea and implications for health seeking behaviour in early infancy – findings from a longitudinal study
Source: BMC Pregnancy Childbirth. 2023 Jan 26;23:71. doi: 10.1186/s12884-022-05322-6 (PMC9878757; doi:10.1186/s12884-022-05322-6)
Supplement: Supplementary file 1 — Additional file 1. [file 12884_2022_5322_MOESM1_ESM.pdf]

Supplementary Tables 1-6:

Supplementary TABLE 1:

Knowledge questions related to newborn danger signs from the interview guide
(available in both English and Tok Pisin)

At ANC
What signs or symptoms in the BABY in the first week after birth do you know that mean you need to go quickly to health care ("Too Sick" Signs)? (choose all mentioned, do not prompt)

At Childbirth
What signs or symptoms in the BABY in the first week after birth do you know that mean you need to go quickly to health care ("Too Sick" Signs)? (choose all mentioned, do not prompt)

At One-month postnatal
What signs or symptoms in the OVER THE NEXT FIVE MONTHS do you know that mean you need to go quickly to health care ("Too Sick" Signs)? (Choose all mentioned, do not prompt.)
All three questions also allowed for 'other comments' by the participants captured as free text.

Supplementary TABLE 2:

Newborn danger signs requiring early intervention

The WHO recommended signs or symptoms were provided as a list to enable interviewers to rapidly code the woman's response to the open questions detailed in the supplementary material above. The Johns Hopkins' Birth Preparedness and Complications Readiness Index indicators were used in data analysis	
**World Health Organisation recommendations on newborn health (2017)**	(1) stopped feeding well,<br>(2) history of convulsions,<br>(3) fast breathing,<br>(4) severe chest in-drawing,<br>(5) no spontaneous movement,<br>(6) temperature >37·5 ºC,<br>(7) temperature <35·5 ºC,<br>(8) any jaundice in first 24 hours of life, or yellow palms and soles at any age.
**Johns Hopkins' Birth Preparedness and Complication Readiness Index (Del Barco, 2004)**	(1) Convulsions/spasms/rigidity<br>(2) Difficult/fast breathing<br>(3) Very small baby<br>(4) Lethargy/unconsciousness

Supplementary TABLE 3:

Other signs reported by the mothers across the three time points

Other signs reported by the mother	Mothers reporting the other signs n (%)		
	**Antenatal Care N = 692**	**Childbirth N = 625**	**One month postnatal N = 581**
**Excessive crying**	158 (22.6)	161 (25.8)	166 (27.7)
**Many skin pustules**	126 (18.0)	110 (17.6)	94 (16.2)
**Cough**	91 (13.0)	129 (20.6)	356 (61.3)
**Baby vomiting**	71 (10.3)	74 (11.8)	88 (15.1)
**Umbilical redness**	63 (9.0)	48 (7.7)	24 (4.1)
**Flu/cold/blocked/runny nose/asthma/sick (URT symptoms)**	57 (8.2)	67 (10.7)	28 (4.8)
**Inability to urinate or stool**	40 (5.7)	62 (9.9)	23 (4.0)
**Diarrhoea/stomach related**	16 (2.3)	36 (5.8)	159 (27.4)
**Eye/ear related**	7 (0.01)	8 (1.3)	8 (1.4)
**Other (* need to list all)**	3 (0.43)	11 (1.8)	11 (1.9)
**Malaria**	3 (0.43)	2 (0.3)	2 (0.3)

Supplementary TABLE 4:

Number of danger signs reported by the mothers at three time points

	Mothers reporting danger signs n (%)		
**Number of WHO Danger Signs Reported by the mother**	**Antenatal Care**<br>**N = 692**	**Childbirth**<br>**N = 625**	**One month postnatal**<br>**N = 581**
None	215 (31.1)	112 (17.9)	87 (15.0)
1	285 (41.2)	298 (47.7)	320 (55.1)
2	134 (19.4)	154 (24.6)	135 (23.2)
3	47 (6.8)	48 (7.7)	32 (5.5)
4	9 (1.3)	11 (1.8)	7 (1.2)
5	2 (0.3)	1 (0.2)	0
6	0	1 (0.2)	0
Missing data:	ANCmissing: 7	DELm: 74	ONEMm: 118

Supplementary TABLE 5:

Association between women naming ≥3 newborn danger signs and potential determinants of knowledge, at interviews at 1st ANC

Sociodemographic and Obstetric characteristics	Crude analysis	Adjusted analysis
	OR (95% CI); p-value	OR (95% CI); p-value
**Enrolment Clinic**		
Vunapope (REF)	REF	REF
Nonga	1.52 (0.63-3.67); 0.354	2.46 (0.97-6.26); 0.059
Keravat	1.18 (0.52-2.7); 0.691	1.32 (0.55-3.2); 0.535
Napapar	1.31 (0.61-2.81); 0.487	2.09 (0.91-4.84); 0.084
Paparatava	0.78 (0.33-1.86); 0.575	1.29 (0.51-3.26); 0.593
**Clinic administration**		
Government (REF)	REF	
Church Health Facility	0.78 (0.44-1.38); 0.398	
**Age, years**		
16-24 (REF)	REF	REF
25-34	1.28 (0.7-2.34); 0.417	1.24 (0.61-2.5); 0.557
35 years or older	1.82 (0.81-4.1); 0.145	1.63 (0.64-4.18); 0.309
**Highest level of education completed**		
Primary school (Grade 8 or less)	REF	REF
High school (Grade 9, 10)	1.13 (0.57-2.26); 0.721	0.68 (0.32-1.45); 0.321
Secondary (Grade 11,12)	2.23 (0.9-5.51); 0.084	1.97 (0.72-5.4); 0.19
Vocational or Tertiary	1.39 (0.7-2.79); 0.349	0.85 (0.4-1.8); 0.666
**Maternal employment status**		
Not employed	REF	
Employed	1.15 (0.60-2.20); 0.666	
**Province of Birth**		
East New Britain	REF	REF
Other Province	**2.37 (1.31-4.31); 0.004**	1.78 (0.91-3.5); 0.092
**Religion**		
Catholic	REF	
United	1.02 (0.55-1.89); 0.96	
Other	1.79 (0.83-3.88); 0.14	
**Household monthly expenditure in Kina**		
Poorest quintile (REF)	REF	REF
50-150	1.14 (0.44-2.97); 0.785	1.08 (0.41-2.9); 0.871
150-300	1.83 (0.75-4.49); 0.186	1.79 (0.7-4.56); 0.225
>300	**4.15 (1.8-9.56); 0.001**	**3.86 (1.53-9.72); 0.004**
**Time to clinic in minutes**		
25mins or less (REF)	REF	
More than 25mins	1.5 (0.84-2.67); 0.173	
**Partner's highest level of education**		
Primary school (Grade 8 or less)	REF	
High school (Grade 9, 10)	1.37 (0.67-2.78); 0.388	
Secondary (Grade 11,12)	1.16 (0.48-2.81); 0.742	
Vocational or Tertiary	0.79 (0.38-1.63); 0.527	
**Partner's employment status**		
Not employed/house duties	REF	REF
Employed	1.82 (1-3.3); 0.05	1.28 (0.66-2.46); 0.464
**Partner present at ANC**		
No not present	REF	REF
Yes at ANC	1.7 (0.91-3.16); 0.097	1.63 (0.85-3.13); 0.145
**Gravidity**		
Primigravida	REF	
Multigravida	1.46 (0.74-2.88); 0.275	1.15 (0.52-2.57); 0.729

NOTE: Marital status (Married or cohabiting/ Single/separated/widowed) was omitted because of collinearity.

Supplementary TABLE 6:

Association between women naming ≥3 newborn danger signs and potential determinants of knowledge, at interviews one month post-partum

Sociodemographic and Obstetric characteristics	Crude analysis	Adjusted analysis
	OR (95% CI); p-value	OR (95% CI); p-value
**Enrolment Clinic**		
Vunapope (REF)	REF	REF
Nonga	0.62 (0.2-1.92); 0.404	0.71 (0.21-2.36); 0.572
Keravat	0.87 (0.36-2.12); 0.76	0.73 (0.26-2.03); 0.545
Napapar	0.44 (0.17-1.15); 0.094	0.48 (0.17-1.38); 0.175
Paparatava	0.38 (0.14-1.06); 0.066	0.52 (0.17-1.58); 0.246
**Clinic administration**		
Government (REF)	REF	
Church Health Facility	0.82 (0.41-1.66); 0.586	
**Age, years**		
16-24 (REF)	REF	REF
25-34	**2.43 (1.07-5.53); 0.034**	2.72 (0.95-7.73); 0.061
35 years or older	**3.24 (1.13-9.32); 0.029**	**3.64 (1.01-13.06); 0.048**
**Highest level of education completed**		
Primary school (Grade 8 or less)	REF	REF
High school (Grade 9, 10)	**3.75 (1.55-9.07); 0.003**	**3.13 (1.2-8.2); 0.02**
Secondary (Grade 11,12)	2.61 (0.66-10.25); 0.171	3.45 (0.8-14.94); 0.098
Vocational or Tertiary	**4.01 (1.62-9.95); 0.003**	**3.09 (1.16-8.22); 0.024**
**Maternal employment status**		
Not employed	REF	
Employed	1.63 (0.79-3.38); 0.188	
**Province of Birth**		
East New Britain	REF	REF
Other Province	**3.38 (1.68-6.79); 0.001**	**2.97 (1.35-6.52); 0.007**
**Religion**		
Catholic	REF	
United	0.73 (0.33-1.58); 0.423	
Other	1.4 (0.54-3.62); 0.491	
**Household monthly expenditure in Kina**		
Poorest quintile (REF)	REF	REF
50-150	0.66 (0.22-1.95); 0.451	0.4 (0.12-1.35); 0.14
150-300	1.32 (0.52-3.4); 0.559	0.99 (0.36-2.76); 0.985
>300	2.19 (0.88-5.41); 0.09	1 (0.35-2.84); 0.998
**Time to clinic in minutes**		
25mins or less(REF)	REF	
More than 25mins	0.89 (0.43-1.85); 0.758	
**Partner's highest level of education**		
Primary school (Grade 8 or less)	REF	
High school (Grade 9, 10)	0.75 (0.27-2.13); 0.59	
Secondary (Grade 11,12)	0.71 (0.19-2.67); 0.611	
Vocational or Tertiary	1.72 (0.77-3.85); 0.187	
**Partner's employment status**		
Not employed/house duties	REF	REF
Employed	1.25 (0.63-2.5); 0.528	0.82 (0.38-1.76); 0.604
**Partner present at ANC**		1.76 (0.8-3.92); 0.162
No not present	REF	
Yes at ANC	1.7 (0.82-3.54); 0.153	
**Gravidity**		
Primigravida	REF	REF
Multigravida	1.45 (0.62-3.36); 0.387	0.81 (0.27-2.44); 0.708

NOTE: Marital status (Married or cohabiting/ Single/separated/widowed) was omitted because of collinearity.
